# Auditory Processing Disorders: Navigating the Diagnostic Maze of Central Hearing Losses

**DOI:** 10.3390/jcm14072256

**Published:** 2025-03-26

**Authors:** Marco Gitto, Noemi Motta, Mirko Aldè, Diego Zanetti, Federica Di Berardino

**Affiliations:** 1 Audiology Unit, Department of Surgical Sciences, Fondazione IRCCS Ca’ Granda Ospedale Maggiore Policlinico, 20122 Milan, Italy; marco.gitto@unimi.it (M.G.); mirko.alde@unimi.it (M.A.); diego.zanetti@unimi.it (D.Z.); federica.diberardino@unimi.it (F.D.B.); 2 Department of Clinical Sciences and Community Health, Dipartimento di Eccellenza 2023–2027, University of Milan, 20122 Milan, Italy

**Keywords:** auditory processing disorder, diagnostic tests, speech perception, auditory pattern, rehabilitation

## Abstract

**Background:** Auditory Processing Disorder (APD) manifests as impaired auditory information processing despite normal peripheral hearing. Current clinical protocols lack standardization, hampering evidence-based intervention development. **Objective:** This review analyzes APD research developments from 2011 to 2025, examining diagnostic criteria, assessment protocols, and treatment effectiveness. **Methods:** Medline, Embase, Scopus, and Cochrane Library databases were analyzed (January 2011–January 2025), following PRISMA guidelines. Two reviewers independently screened 413 articles, with 156 meeting inclusion criteria. Analysis included chi-square tests for intervention distribution and *t*-tests for diagnostic comparisons (α = 0.05). **Results:** Among 156 studies analyzed, medical interventions were markedly underrepresented (n = 4) compared to rehabilitative approaches (n = 52; χ^2^ = 50.28, *p* < 0.001). The Random Gap Detection Test and Dichotic Digits Difference Test were most frequently used (12.86% and 10.48% of studies, respectively). Cognitive skill enhancement dominated intervention strategies (52.8%), followed by computer-based rehabilitation (26.4%). Publication frequency showed consistent annual growth, peaking at 57 studies in 2024. Sample sizes were comparable between APD and CAPD studies (mean difference = 4.2 cases, t = 0.416, *p* = 0.679). Environmental modifications appeared in 15.1% of interventions, while speech therapy was reported in only 3.8% of studies. **Conclusions:** The substantial imbalance between medical and rehabilitative interventions necessitates standardized diagnostic protocols and enhanced multidisciplinary collaboration. Implementation of a hierarchical processing framework is recommended for assessment and treatment. Future research should prioritize large-scale controlled trials and unified diagnostic criteria development.

## 1. Introduction

Auditory Processing Disorder (APD) has been a subject of significant research interest for decades, with numerous authors proposing various approaches to both assessment and treatment. Methodologies, particularly in the pediatric population, have been extensively investigated. APD manifests as impaired auditory information processing despite intact peripheral hearing, leading to significant comprehension challenges, especially evident in learning tasks. This disorder particularly affects the ability to process and interpret acoustic signals, directly affecting academic performance and educational outcomes [1,2].

The current clinical management of APD remains challenging for healthcare professionals, primarily due to the absence of standardized diagnostic protocols. The lack of shared guidelines between speech therapy and audiology has resulted in heterogeneous diagnostic approaches that complicate the treatment selection. This diagnostic variability presents a significant barrier to the establishment of evidence-based intervention protocols [3].

A systematic review by Fey et al. [4] evaluated intervention strategies for children and adolescents with APD, examining treatments that target spoken and written language deficits alongside fundamental auditory processing impairments. Their analysis provided insights into available therapeutic approaches while identifying knowledge gaps in treatment efficacy.

The present systematic review extends the work of Fey et al. [4] by analyzing developments in APD research throughout January 2025. We evaluated recent medical and rehabilitative intervention advances, examining emerging diagnostic criteria and assessment protocols. This analysis aims to synthesize current evidence regarding evaluation methodologies and treatment effectiveness in pediatric APD populations, addressing the evolving landscape of diagnostic and therapeutic approaches over the past decade.

## 2. Materials and Methods

A systematic review was conducted with the help of a medical librarian across all Medline and Embase libraries (including the PubMed databases) to identify studies that detail the identification and selection criteria of APD and its diagnostic, therapeutic, and rehabilitative approaches. Studies published from January 2011 to January 2025 were included to capture recent practices. A comprehensive search was applied to additional databases (Scopus and Cochrane Library). Gray literature sources, such as conference proceedings, thesis repositories, and book chapters, were excluded to ensure the inclusion of peer-reviewed studies. The query strings were developed as described in Table 1.

### 2.1. Inclusion and Exclusion Criteria

In order to be included, the selected studies had to be published in peer-reviewed journals; contain original data on identification and/or selection criteria; address diagnostic, therapeutic, and/or rehabilitative approaches for APD; focus on participants diagnosed with “APD” or “CAPD” (central auditory processing disorders); and cover various age groups and populations.

Excluded studies were those involving participants with autism, hearing loss, or cognitive disabilities (IQ < 70), unless comorbid with APD; and articles not written in English.

**Figure 1:**
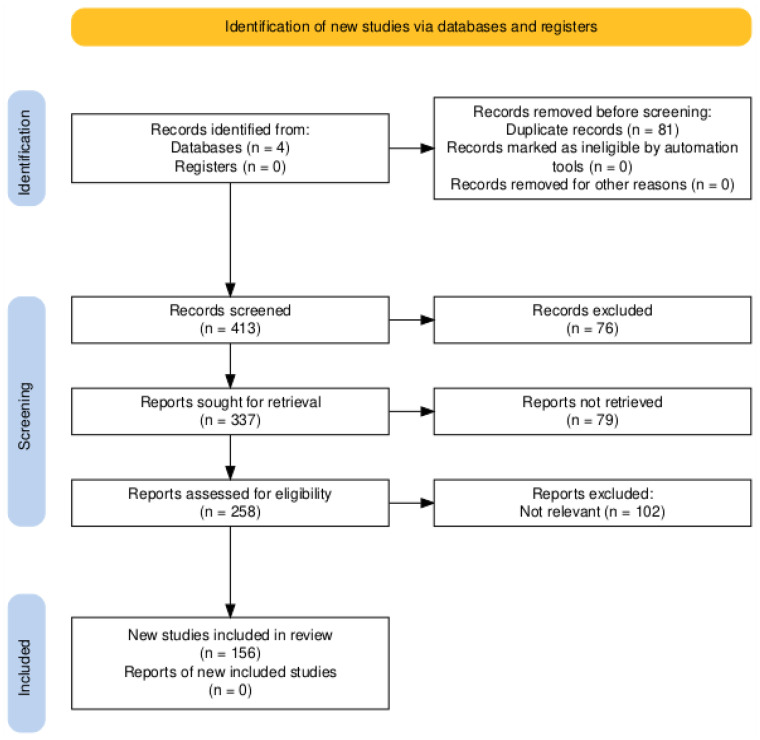
PRISMA flow diagram illustrating the selection process, resulting in the inclusion of 156 studies after screening and eligibility assessments.

All citations were collected and uploaded into Zotero (2021, Vienna, VA, USA: Corporation for Digital Scholarship), and duplicates were removed. Titles and abstracts were screened independently by two reviewers against the inclusion criteria. The full-text articles of potentially relevant studies were assessed in detail. Any disagreements were resolved through discussion. The review was performed in full accordance with PRISMA guidelines (Preferred Reporting Items for Systematic Review and Meta-Analyses) [5]. The selection process was documented using a PRISMA flow diagram (Figure 1).

### 2.2. Data Extraction

Data, extracted independently by two reviewers, included the following:

- Study characteristics: author(s), year, study design;
- Participant details: age, gender, diagnosis (APD or CAPD), and comorbid conditions;
- Diagnostic approaches: tools and methods used;
- Therapeutic and rehabilitative interventions: type, duration, and measured outcomes;
- Key findings relevant to the review questions.

Study authors were contacted for clarification of missing or unclear data.

### 2.3. Data Analysis

An approach combining descriptive and inferential statistics was followed. Descriptive statistics were calculated for publication years (means, standard deviations, and medians) and study characteristics (frequencies and percentages). The temporal distribution of publications was analyzed using frequency counts per year, with central tendency measures calculated to assess publication patterns across the study period (2011–2025).

Statistical analyses included chi-square tests to examine the relationship between treatment approaches and study characteristics (χ^2^ test, α = 0.05). Independent samples’ *t*-tests were conducted to compare case numbers between APD and CAPD diagnostic groups. Pearson correlation coefficients were calculated to assess the relationship between publication year and sample sizes. For all inferential statistical tests, significance was set at *p* < 0.05. Categorical data, including study types and diagnostic classifications, were analyzed using frequency distributions and percentages. The analysis of medical and rehabilitation interventions was conducted through frequency counts and chi-square tests to assess the distribution of treatment approaches across studies. All statistical analyses were performed using SPSS version 28.

## 3. Results

The results of this systematic review are illustrated in Figure 1.

Articles were extracted from four databases. During the initial screening, 81 articles were eliminated due to duplication. In the selection phase, 413 articles were examined across the four databases, of which 76 were excluded because the title and abstract were not relevant. Of the 337 reports sought for retrieval, 79 were not retrieved as they were deemed irrelevant after abstract review. Among the 258 articles that underwent full-text evaluation, 156 were deemed suitable, while 102 were excluded due to the lack of relevance assessed via the reading of the abstract text (topic not pertinent). The dataset encompassed various study types, with observational studies (n = 40), literature reviews (n = 23), cross-sectional studies (n = 13), and systematic reviews (n = 11) being the predominant methodological approaches. An analysis of diagnostic terminology revealed that APD was the primary diagnosis in 98 studies, while Central Auditory Processing Disorder (CAPD) was specified in 39 studies. A *t*-test comparing case numbers between APD and CAPD studies showed no significant difference (t = 0.416, *p* = 0.679), indicating consistency in sampling approaches, regardless of terminology. Publication frequency analysis revealed an upward trend across the study period (2011–2025), with a notable peak in 2024 (57 publications). The mean publication year was 2018 (SD = 3.24), with the median falling in 2019, indicating a slight positive skew in the temporal distribution of publications. An analysis of treatment approaches revealed a minimal implementation of medical therapies, with only 4 studies reporting medical interventions compared to 138 studies reporting no medical therapy (χ^2^ = 126.76, *p* < 0.001). Rehabilitation interventions were more frequently documented, with 52 studies implementing rehabilitation protocols compared to 102 studies reporting no rehabilitation services. The analysis reveals a disparity between medical and rehabilitative interventions, with a minimal implementation of medical therapies (n = 4) compared to rehabilitation protocols (n = 52; χ^2^ = 50.28, *p* < 0.001). Case numbers across studies showed considerable variation (range: 0–1113; M = 106.45, SD = 182.22, median = 50). A *t*-test comparing case numbers between APD and CAPD studies showed no significant difference (t = 0.416, *p* = 0.679), indicating similar sampling approaches, regardless of diagnostic terminology. Correlation analysis between publication year and case numbers revealed no significant relationship (r = −0.053, *p* = 0.632), suggesting that sample sizes remained relatively stable across the study period. An analysis of study methodologies revealed a predominance of observational approaches, followed by literature reviews and cross-sectional designs. A descriptive framework that is still actual and clarifying the topodiagnostical process, by dividing the APD according to the site of lesion, was reported by Gail J. Richard [6]; it is described in Table 2.

### 3.1. APD Tests

An analysis of the methods reported in the reviewed articles revealed distinct patterns in the use of auditory processing tests, as reported in Table 3.

Among the studies, the most frequently employed tests were the Random Gap Detection Test (RGDT) [7] and the Dichotic Digits Difference Test (DDdT) [8], used in 12.86% and 10.48% of studies, respectively. These tests are used for assessing temporal resolution and binaural integration, core components of auditory processing. The Pitch Pattern Sequence (PPS) Test [9] and Frequency Pattern Test (FPT) [10] were each mentioned in 7.38% of studies. These tests evaluate auditory pattern temporal ordering and pitch discrimination—core components of phonetic–phonological processing. Similarly, the Duration Pattern Sequence (DPS) Test [9] was utilized in 4.29% of studies, which is used for assessing the timing aspects of auditory processing. The Listening in Spatialized Noise—Sentences Test (LiSN-S) [11] appeared in 4.05% of studies. This test evaluates spatial processing and speech-in-noise understanding. The Gaps In Noise (GIN) Test [12], which assesses temporal resolution in challenging listening conditions, was cited in only 2.86% of the reviewed articles.

**Table 3:** Key characteristics of auditory processing disorder tests (modified from Geffner and Ross-Swain (2018) [13]).

Test Name	First Author and Year	Age Range	Description	Auditory Processing Skill Assessed	Sensitivity to Lesions/Disorders	Utility in APD Evaluation
Pitch Pattern Sequence (PPS) Test	Musiek, F.E., 1994 [9]	Ages 9 and up	Sequences of three tone bursts at two different frequencies presented monaurally.	Auditory pattern temporal ordering (APTO); temporal sequencing abilities.	Sensitive to cortical lesions and interhemispheric transfer issues.	Assesses temporal sequencing crucial for understanding prosodic elements of speech; uses non-verbal stimuli to eliminate language influence.
Random Gap Detection Test (RGDT)	Keith, R. W., 2000 [7]	Ages 5 and up	Pairs of tones with varying silent intervals; listener detects presence of gaps.	Temporal resolution.	Sensitive to cortical lesions, particularly left temporal lobe.	Measures ability to perceive brief gaps essential for processing rapid acoustic changes in speech; includes age-appropriate norms.
Gaps In Noise (GIN) Test	Musiek, F. E., 2005 [12]	Ages 7 and up	Noise bursts containing silent intervals (gaps) of varying durations; listener detects gaps within noise.	Temporal resolution in complex listening conditions.	Evaluates central auditory processing; does not address specific lesions.	Assesses gap detection in noisy backgrounds, mirroring real-life listening situations; important for decoding speech in noise; includes normative data.
Duration Pattern Sequence (DPS) Test	Musiek, F. E., 1994 [9]	Ages 11 and up	Sequences of three tones at a single frequency with two different durations (short and long); listener identifies temporal pattern based on duration.	Auditory pattern temporal ordering (APTO); temporal ordering skills.	Sensitive to cortical lesions and interhemispheric transfer problems.	Evaluates ability to recognize and sequence temporal patterns, important for processing timing aspects of speech; non-verbal assessment suitable for diverse populations.
Frequency Pattern Test (FPT)	Pinheiro, M. L., 1971 [10]	Ages 8 and up	Sequences of three tone bursts at two different frequencies presented monaurally.	Auditory pattern temporal ordering (APTO); pitch discrimination.	Sensitive to cortical lesions and interhemispheric transfer deficits.	Tests ability to discriminate between high and low frequencies in a sequence, essential for recognizing speech intonation and melody; broad age applicability.
Listening in Spatialized Noise—Sentences Test (LiSN-S)	Cameron, S., 2009 [11]	Ages 6 and over	Measures speech understanding in noise using spatialized audio to create a 3D auditory environment; 4 subtests assess SNR for 50% sentence comprehension.	Spatial processing; speech-in-noise understanding.	Detects spatial processing disorder (SPD); common after otitis media in early childhood.	Assesses ability to use spatial cues to segregate sounds; simulates real-life listening conditions; provides functional assessment data; extensive normative data included.
Dichotic Digits Difference Test (DDdT)	Cameron, S., 2016 [8]	Ages 7 and over	Different digit names presented simultaneously to each ear (dichotic) and same digits to both ears (diotic); includes free recall and directed ear conditions.	Binaural integration; dichotic listening skills.	Differentiates cognitive deficits from dichotic processing issues.	Tests ability to process different auditory information presented to both ears simultaneously; includes control condition to distinguish auditory from cognitive deficits; provides derived scores like right-ear advantage.

### 3.2. Training and Intervention Strategies for Auditory Processing Disorders

A statistical analysis of the included papers, incorporating broader search keys and alternative terms, provides a comprehensive view of the training and intervention strategies described in the reviewed studies. Seven primary categories were identified, each reflecting a unique approach to managing APDs.

#### 3.2.1. Auditory Rehabilitation Therapy

This category was underrepresented, appearing in only one study (1.9% of the total). Auditory Discrimination Training, Listening Exercises, and Temporal Processing Enhancement were occasionally referenced as the main methods to address auditory deficits but lacked widespread documentation.

#### 3.2.2. Environmental Modifications

Environmental interventions were noted in eight studies (15.1%), making them a moderately represented category. Acoustic Enhancements (e.g., FM systems, amplifiers, sound-absorbing panels) and Communication Strategies to improve auditory comprehension in noisy environments were the most common approaches.

#### 3.2.3. Cognitive Skill Enhancement

This was the most frequently cited category, reported in 28 studies (52.8%). Working Memory Training and Selective Attention Techniques were prominently featured, targeting cognitive abilities that underpin effective auditory processing.

#### 3.2.4. Computer-Based Auditory Rehabilitation Programs

Referenced in 14 studies (26.4%), this category included programs such as Fast ForWord, Earobics, and LACE (Listening and Communication Enhancement). These programs leverage interactive, gamified exercises to enhance auditory discrimination and temporal processing.

#### 3.2.5. Speech Therapy

This category, in its general definition, appeared in only two studies (3.8%). Interventions focusing on Articulation and Pronunciation Training and Verbal Comprehension Therapy were minimally discussed.

#### 3.2.6. Educational Interventions

No studies explicitly addressed educational accommodations such as Classroom Adaptations or Assistive Technologies.

#### 3.2.7. Multisensory Therapies

Similarly, no studies mentioned Sensory Integration Approaches combining auditory, visual, and tactile modalities.

### 3.3. Intervention Distribution

A statistical evaluation of the intervention distribution revealed significant differences among the categories. A chi-square goodness-of-fit test showed a chi-square statistic of 85.55 (*p* < 0.001), confirming that the observed distribution of intervention strategies deviates significantly from a uniform distribution.

#### 3.3.1. Types of Intervention

Cognitive Skill Enhancement and Computer-Based Programs were significantly overrepresented, comprising 52.8% and 26.4% of the total interventions, respectively. In contrast, Speech Therapy, Educational Interventions, and Multisensory Therapies were underrepresented.

#### 3.3.2. Pharmacological Treatment

Pharmacological treatment was administered in three studies [14,15,16].

##### Anti-Inflammatory and Immunosuppressive Agents

Prednisone was employed in one study, analyzing three cases [14]. Its application ranged from standalone use to combinations with anticonvulsants and psychotropic medications.

##### Anticonvulsants and Neuromodulators

One study reported the use of anticonvulsants such as Sulthiame, Carbamazepine, and Vigabatrin. These agents were utilized to stabilize neural excitability and manage seizure-like symptoms, often in combination with Prednisone [14].

##### Psychotropic Medications

One study examined the use of Citalopram, a selective serotonin reuptake inhibitor (SSRI), often used to manage comorbid depressive symptoms in APD populations [15].

##### Central Nervous System Stimulants

Methylphenidate was employed in one study to enhance attentional processes and support auditory processing improvements, demonstrating the relevance of cognitive enhancement strategies in APD management [16].

While valuable in addressing inflammation, neural excitability, and comorbid psychological symptoms, pharmacological treatment is not sufficient as a standalone intervention for auditory processing disorders. Its role appears to be primarily supportive, complementing more foundational strategies such as environmental modifications and resource-based interventions. This integrated approach aligns with the multifactorial nature of APDs, emphasizing the necessity of addressing both the clinical and contextual factors underlying auditory processing challenges.

## 4. Discussion

This review reveals significant trends in the research on APD from 2011 to 2025. The analysis of 156 studies demonstrates increased research attention, as evidenced by a publication peak in 2024. This reflects a growing recognition of APD’s importance in clinical practice.

On the other hand, terminological variation between APD and CAPD designations (98 vs. 39 studies), for example, reflects ongoing debate about diagnostic classification. However, our finding of no significant difference in case numbers between APD and CAPD studies indicates consistency in methodological approaches, regardless of terminology.

Several questions persist about the number, type, degree, and combination of defective processing skills that can be grouped under the diagnosis of APD and which abilities warrant priority in assessment. The absence of standardized test batteries has led to variability in clinical practice, with audiologists, speech and language pathologists, and neurologists developing assessment protocols that are based on clinical observations from different points of view.

The literature demonstrates the underrepresentation of standardized auditory processing tests. Despite the established utility of tests such as the Pitch Pattern Test, Random Gap Detection Test, and Frequency Pattern Test, as suggested by Geffner and Ross-Swain (2018) [13], only one study in our Embase dataset referenced the Frequency Pattern Test. This suggests either a significant research gap or challenges in search methodology related to test nomenclature.

The complexity of APD assessment is intensified by the relationship between auditory processing and language skills. Language deficits significantly impact auditory processing test performance, creating a vicious loop with the existing diagnostic challenges. For example, semantic language deficits could affect the comprehension of dichotic listening test instructions [17], potentially leading to diagnostic uncertainty, particularly in cases with comorbid conditions such as language disorders [17], attention deficit hyperactivity disorder [18], or autism spectrum disorders [19].

Our work supports the framework proposed by Gail J. Richard [6], emphasizing three hierarchical levels of auditory processing: acoustic processing, phonetic–phonological processing, and linguistic–communicative processing. Rather than addressing all levels indiscriminately, the approach advocates for targeted interventions based on the specific processing deficits that are identified in any single patient.

### 4.1. Acoustic Processing

At the peripheral auditory system and brainstem level, interventions aim to ensure accurate signal reception and transmission. Addressing hearing loss or central auditory signal distortions is typically managed by audiologists, who will set appropriate acoustic amplification and environmental signal-to-noise improvements. Possible developments involve a targeted training of higher-order auditory processing by trained speech therapists.

### 4.2. Phonetic—Phonological Processing

This stage bridges the auditory and linguistic domains, because it involves the transverse temporal gyri. The interventions focus on phonological awareness and phonetic discrimination, often requiring collaboration between audiologists and speech–language pathologists for tasks like pattern recognition and segmentation training.

### 4.3. Linguistic—Communicative Processing

This stage encompasses the temporal, frontal, and prefrontal cortices, where linguistic interpretation and meaning extraction occur. Interventions focus on semantic and pragmatic language skills, typically delivered by speech–language pathologists, who aim at integrating the auditory input into functional communication.

Adopting this structured framework ensures that interventions are precise and aligned with the specific needs of each processing level, thus maximizing the therapeutic efficacy.

Current evidence supports a processing continuum from basic acoustic reception to complex linguistic interpretation. Richard’s model [6], described in Table 2, emphasizes the importance of collaboration between audiologists and speech–language pathologists, especially in evaluating phonetic–phonological processing, where their expertise intersects.

At the moment, a persistent debate is centered on intervention focus: whether to target discrete auditory skills or functional auditory processing. While evidence indicates improvement in specific auditory skills following an intervention, our findings, extending Fey et al.’s work [4], show limited generalization to academic and language gains.

Effective treatment for APD necessitates collaboration not only between audiologists and speech–language pathologists, but also with neurologists, psychologists, pediatricians, and rehabilitation therapists. The latter should be involved in the case of concurrent neuro-motor disturbances (i.e., dysarthrias). Audiologists play a key role in diagnosis and auditory rehabilitation therapy, focusing on signal reception and transmission. Speech–language pathologists address deficits in phonetic–phonological and linguistic–communicative processing, while neuropsychologists assess cognitive functions and develop strategies to support attention, memory, and executive processes. Psychologists provide critical emotional and behavioral support, particularly for children with comorbid conditions, and medical professionals oversee pharmacological management and address related medical concerns.

In addition to professional contributions, the roles of teachers, educators, and parents are essential in APD management. Teachers and educators adapt learning strategies and environments to the specific needs of the child, while parents provide a consistent reinforcement of therapeutic strategies and serve as advocates for their child’s educational and clinical needs. The coordination among these stakeholders is essential for achieving an effective and personalized treatment plan [6].

Several limitations of the present systematic review warrant consideration. The exclusion of gray literature may have omitted some findings from conference proceedings and unpublished studies. Heterogeneous diagnostic criteria and outcome measures complicated the effectiveness of the comparison among different interventions. Additionally, the validity of some assessment tools remains questioned [20], indicating a need for more robust validation studies.

## 5. Conclusions

This review provides an analysis of APD research developments from 2011 to 2025, highlighting significant progress and persistent challenges. The growing research attention, particularly evident in 2024, underscores the increasing recognition of APD’s importance in clinical and academic contexts.

The findings of this review reveal an imbalance between medical and rehabilitative interventions. Terminological inconsistencies persist across studies. The predominance of observational studies indicates a need for more interventional research designs to strengthen the evidence base. Additionally, the limited documentation of standardized auditory processing tests in the literature, despite their demonstrated clinical efficacy, highlights a gap that warrants attention.

Looking forward, some research priorities emerge: large-scale controlled trials of rehabilitation interventions, investigation of medical therapy roles, standardization of diagnostic criteria and terminology, and systematic evaluation of standardized auditory processing test outcomes.

## Figures and Tables

**Table 1:** Query strings developed for the Medline/PubMed, Embase, Scopus, and Cochrane Library databases.

Database	Query String
Medline/PubMed	((“central auditory processing disorder”[Title/Abstract]) OR (“auditory processing disorder”[Title/Abstract])) AND ((treatment[Title/Abstract]) OR (diagnosis[Title/Abstract]) OR (intervention[Title/Abstract]))
Embase	(‘central auditory processing disorder’/exp OR ‘central auditory processing disorder’ OR ‘auditory processing disorder’/exp OR ‘auditory processing disorder’) AND (‘treatment’ OR ‘treatment’/exp OR treatment OR ‘diagnosis’ OR ‘diagnosis’/exp OR diagnosis OR ‘intervention’ OR ‘intervention’/exp OR intervention) AND ([cochrane review]/lim OR [controlled clinical trial]/lim OR [systematic review]/lim OR [randomized controlled trial]/lim OR [meta analysis]/lim) AND [2011–2025]/py
Scopus	TITLE-ABS-KEY (“central auditory processing disorder*” OR “auditory processing disorder*”) AND TITLE-ABS-KEY (“treat*” OR “therap*” OR “intervention*” OR “rehabilitat*”) AND PUBYEAR > 2010 AND PUBYEAR < 2026
Cochrane Library	((“central auditory processing disorder*” OR “auditory processing disorder*”)):ti,ab,kw AND ((treat* OR therap* OR intervention* OR rehabilitat*)):ti,ab,kw (Date published on the Cochrane Library: Between Jan 2011 and Jan 2025)

**Table 2:** Hierarchical organization framework of auditory and language processing (categorized by Gail J. Richard [6]).

Component	Anatomical Structures	Type of Processing
Peripheral auditory system	Outer ear, middle ear, inner ear	Auditory acuity, signal reception
Central auditory processing	CNS, brainstem	Signal transfer; discrimination of the signal’s acoustic characteristics
Phonetic-phonological processing	Transverse temporal gyri (Heschl’s convolutions), temporal cortex	Discrimination of the phonetic-phonological characteristics of the signal
Language processing	Temporal cortex, Wernicke’s area, angular gyrus	Discrimination of the linguistic characteristics of the signal, assigning meaning through the linguistic code
Executive functions	Frontal and prefrontal cortex, primary motor cortex	Planning and execution of the response

## Data Availability

The review has not been registered on an appropriate database (e.g., PROSPERO). The data will be provided upon request.
